# Neurophysiology of space travel: energetic solar particles cause cell type-specific plasticity of neurotransmission

**DOI:** 10.1007/s00429-016-1345-3

**Published:** 2016-11-30

**Authors:** Sang-Hun Lee, Barna Dudok, Vipan K. Parihar, Kwang-Mook Jung, Miklós Zöldi, Young-Jin Kang, Mattia Maroso, Allyson L. Alexander, Gregory A. Nelson, Daniele Piomelli, István Katona, Charles L. Limoli, Ivan Soltesz

**Affiliations:** 1 Department of Anatomy and Neurobiology, University of California, Irvine, CA 92697 USA; 2 Department of Radiation Oncology, University of California, Irvine, CA 92697 USA; 3 Department of Neurosurgery, and Neurology and Neurological Sciences, Stanford University, Palo Alto, CA 94305 USA; 4 Momentum Laboratory of Molecular Neurobiology, Institute of Experimental Medicine, Hungarian Academy of Sciences, 1083 Budapest, Hungary; 5 Department of Neurology, University of Arkansas for Medical Sciences, Little Rock, AR 72205 USA; 6 School of Ph.D. Studies, Semmelweis University, Budapest, Hungary; 7 Division of Radiation Research, Department of Basic Sciences, Loma Linda University, Loma Linda, CA 92350 USA

**Keywords:** Irradiation-induced cognitive impairments, GABAergic interneurons, Cannabinoid signaling system

## Abstract

In the not too distant future, humankind will embark on one of its greatest adventures, the travel to distant planets. However, deep space travel is associated with an inevitable exposure to radiation fields. Space-relevant doses of protons elicit persistent disruptions in cognition and neuronal structure. However, whether space-relevant irradiation alters neurotransmission is unknown. Within the hippocampus, a brain region crucial for cognition, perisomatic inhibitory control of pyramidal cells (PCs) is supplied by two distinct cell types, the cannabinoid type 1 receptor (CB_1_)-expressing basket cells (CB_1_BCs) and parvalbumin (PV)-expressing interneurons (PVINs). Mice subjected to low-dose proton irradiation were analyzed using electrophysiological, biochemical and imaging techniques months after exposure. In irradiated mice, GABA release from CB_1_BCs onto PCs was dramatically increased. This effect was abolished by CB_1_ blockade, indicating that irradiation decreased CB_1_-dependent tonic inhibition of GABA release. These alterations in GABA release were accompanied by decreased levels of the major CB_1_ ligand 2-arachidonoylglycerol. In contrast, GABA release from PVINs was unchanged, and the excitatory connectivity from PCs to the interneurons also underwent cell type-specific alterations. These results demonstrate that energetic charged particles at space-relevant low doses elicit surprisingly selective long-term plasticity of synaptic microcircuits in the hippocampus. The magnitude and persistent nature of these alterations in synaptic function are consistent with the observed perturbations in cognitive performance after irradiation, while the high specificity of these changes indicates that it may be possible to develop targeted therapeutic interventions to decrease the risk of adverse events during interplanetary travel.

**Electronic supplementary material:**

The online version of this article (doi:10.1007/s00429-016-1345-3) contains supplementary material, which is available to authorized users.

## Introduction

Deep space travel poses variety of challenges to humankind, including an unavoidable exposure to unique radiation fields that present a range of health risks (Cucinotta et al. 2014). These fields consist of both charged particles including energetic protons ejected from the sun during solar particle events (SPEs) and galactic cosmic rays comprised of fully ionized atomic nuclei emitted from sources beyond our solar system (Nelson 2016). Recent evidence in rodents demonstrates that exposure to such radiation types can elicit persistent impairments in cortical and hippocampal based learning and memory (Britten et al. 2012; Lonart et al. 2012). These findings are a potential concern to NASA, as astronauts operating under increased autonomy may be at heightened risk for manifesting performance-based decrements that could compromise mission critical activities, or for developing long-term neurocognitive complications. Cognitive changes can be linked to structural alterations in specific neuronal subsets, involving reduced dendritic complexity and spine density and alterations in the distribution and levels of critical synaptic proteins (Lonart et al. 2012; Parihar and Limoli 2013; Sweet et al. 2014; Allen et al. 2015; Chmielewski et al. 2016). Despite these findings, relatively little is known regarding the impact of charged particles on specific excitatory and inhibitory circuits in the brain.

Inhibitory GABAergic interneurons and excitatory cells form highly cell type-specific neuronal circuits throughout the cortical mantle which generate functionally important network activity (Yoshimura and Callaway 2005; Isaacson and Scanziani 2011). In particular, GABAergic interneurons in the hippocampus are known to underlie the emergence of theta and gamma oscillations (Klausberger and Somogyi 2008; Isaacson and Scanziani 2011; Hu et al. 2014). These rhythms play key roles in spatial memory, attention, and cognitive flexibility (Fuchs et al. 2007; Basu et al. 2013; Cho et al. 2015; Hanslmayr et al. 2016; Kim et al. 2016), which are behavioral tasks known to be impaired after low-dose charged particle irradiation (Britten et al. 2012; Lonart et al. 2012; Parihar et al. 2014; Bellone et al. 2015). Because space relevant exposures elicit cognitive deficits involving impaired hippocampus-dependent spatial memory (Britten et al. 2012; Bellone et al. 2015; Parihar et al. 2015a), irradiation might compromise hippocampal GABAergic interneurons and/or their connectivity with excitatory cells.

Among functionally distinct subtypes of GABAergic interneurons (Klausberger and Somogyi 2008), cholecystokinin and cannabinoid type 1 receptor (CB_1_)-expressing, regular-spiking basket cells (CB_1_BCs) and parvalbumin-expressing, fast-spiking interneurons (PVINs) are two non-overlapping classes of cells which together provide the entirety of perisomatic inhibition to principal cells (Katona et al. 1999; Armstrong and Soltesz 2012; Bezaire and Soltesz 2013). These powerful inhibitory cells regulate normal cortical network activity and have also been implicated in neurological disorders including epilepsy, autism, and schizophrenia (Chen et al. 2007; Armstrong and Soltesz 2012; Curley and Lewis 2012; Földy et al. 2013). Given the distinct roles of CB_1_BCs and PVINs in circuit operations (Armstrong and Soltesz 2012), we hypothesized that energetic protons characteristic of cosmic rays and SPEs might compromise the microcircuits involving these key GABAergic interneurons. Mice subjected to whole body proton irradiation to realistically simulate solar exposures were analyzed 2 months later for disruptions to hippocampal circuitry. These results demonstrate that low dose proton irradiation selectively modulates hippocampal microcircuits.

## Materials and methods

All experiments were conducted in accordance with the Institutional Animal Care and Use Committee of the University of California, Irvine, Stanford University, the University of Arkansas for Medical Sciences, and Loma Linda University, as well as according to Hungarian Act of Animal Care and Experimentation (1998, XXVIII, Section 243/1998), which are in accordance with the European Communities Council Directive of November 24, 1986 (86/609/EEC; Section 243/1998).

### Animals

To target PVINs for patch-clamp recordings, a PV-Cre line (The Jackson Laboratory stock # 008069) was crossed with a reporter line (The Jackson Laboratory stock # 007905) to produce mice expressing the red fluorescent protein tdTomato in PV + cells (PV-TOM mice). C57BL/6J mice (The Jackson Laboratory stock # 000664) were used in all other experiments.

### Radiation exposure

Male C57BL/6J or PV-TOM mice at 2–3 months of age were exposed to whole body 150 MeV/n proton irradiation, and the irradiated group received 0.502 ± 0.004 Gy proton irradiation at a dose rate of 0.75 ± 0.06 Gy/min. Irradiation was performed at the James M. Slater Proton Therapy Treatment and Research Center (Loma Linda University Medical Center, Loma Linda, CA). Due to scattering foils and other beamline components, the proton energy at the target surface was reduced to 126 MeV and was associated with an LET of 0.62 keV/μm. Control mice were identically treated except that they were not exposed to proton irradiation.

### In vitro electrophysiology

Coronal hippocampal slices (300 μm) were prepared from male PV-TOM mice or C57BL/6J mice 5–9 weeks after irradiation or sham treatment. Slices were incubated in sucrose-containing artificial CSF (ACSF) for an hour. ACSF contained, in mM (85 NaCl, 75 sucrose, 2.5 KCl, 25 glucose, 1.25 NaH_2_PO_4_, 4 MgCl_2_, 0.5 CaCl_2_, and 24 NaHCO_3_). After the initial incubation period, slices were transferred in the same ACSF solution used for recordings, which contained, in mM (126 NaCl, 2.5 KCl, 26 NaHCO_3_, 2 CaCl_2_, 2 MgCl_2_, 1.25 NaH_2_PO_4_, and 10 glucose). Patch pipettes had resistances of 3–5 MΩ. Signals were filtered at 3 kHz using a Bessel filter and digitized at 10 kHz with a Digidata 1440A analog–digital interface (Molecular Devices). Series resistances were carefully monitored, and recordings were discarded if the series resistance changed >20% or reached 20 MΩ. The recorded traces were analyzed using Clampfit 10.5 (Molecular Devices). Slices were visualized with an upright microscope (Olympus; BX61WI) with infrared–differential interference contrast (IR-DIC) optics. These microscopes were additionally equipped with a mercury lamp light source for epifluorescence. All electrophysiological recordings were made at 33 °C using a MultiClamp700B amplifier (Molecular Devices).

To examine interneuron to PC connections, whole-cell recordings in current-clamp configuration were obtained from the CB_1_BCs or PVINs with Vm adjusted to −60 mV. The interneuronal internal solution contained (in mM) 126 K-gluconate, 4 KCl, 10 HEPES, 4 ATP-Mg, 0.3 GTP-Na, 10 phosphocreatine, as well as 0.2% biocytin, with a pH of 7.2, and osmolarity of 290 mOsm. Superficial PCs were recorded in voltage-clamp at a holding potential of −70 mV. The PC internal solution contained (in mM) 40 CsCl, 90 K-gluconate, 1.8 NaCl, 1.7 MgCl_2_, 3.5 KCl, 0.05 EGTA, 10 HEPES, 2 Mg-ATP, 0.4 Na_2_GTP, and 10 phosphocreatine as well as 0.2% biocytin, with a pH 7.2, and osmolarity of 290 mOsm. Action potentials in presynaptic interneurons were induced in current clamp by injecting 2 ms square pulses of 2nA. In a subset of pairs, we tested the PC to interneuron connections as well (PC, voltage-clamp, holding potential, −70 mV; interneurons, voltage-clamp, holding potential, −60 mV). Action currents in presynaptic PCs were induced in voltage-clamp by injecting 2 ms square pulses from −70 to 30 mV.

To examine the intrinsic properties of CB_1_BCs or PVINs, we recorded interneurons using an internal solution containing (in mM) 126 K-gluconate, 4 KCl, 10 HEPES, 4 m ATP-Mg, 0.3 GTP-Na, 10 phosphocreatine, as well as 0.2% biocytin, with a pH 7.2, and osmolarity of 290 mOsm. Interneurons were held at their resting membrane potentials. Action potential discharges were evoked by current injections (1 s-long step currents, from 0 to +500 pA with 50 pA increments). To determine the input resistance of interneurons, they were held at their resting membrane potentials and voltage responses to small current pulses (1 s-long current steps from −100 to +100 pA with 50 pA increments) were measured at steady state (0.8–1.0 s from the start of 1 s-long current steps).

### Cellular identification

For recordings from presynaptic CB_1_BCs, multipolar neurons located in the striatum radiatum were targeted using infrared differential interference contrast microscopy and filled with biocytin for post hoc cell identification. All putative CB_1_+ interneurons were identified post hoc as CB_1_BCs by their characteristic axonal arborization and histological immunopositivity for CB_1_ (see below for details). PVINs were targeted based on tdTomato fluorescence. The somata of all recorded CA1 PCs were located within the superficial sublayer of the stratum pyramidale (distance from the pyramidale-radiatum border: 0–20 μm, dorsal hippocampus; 0–50 µm, ventral hippocampus; see Lee et al. 2014 for the rationale and discussion of the superficial sublayer).

### Reconstruction of CB_1_BCs and analysis of their axonal/dendritic morphology

CB_1_BCs were filled with biocytin in hippocampal slices from C57BL/6J mice a mean of 8 weeks (range 7–9 weeks) post-irradiation. Slices were resectioned into 40–70 μm thin sections. Cells not processed for STORM imaging, were processed for CB_1_ immunopositivity (CB1-GP-Af530-1; 1:5000, guinea pig; Frontiers Science). A secondary antibody conjugated to Alexa Fluor 488, raised in donkey against guinea pig (Invitrogen), was used to detect the location of the primary antibody. Biocytin was visualized DyLight594-labeled streptavidin (Jackson, 1:1000). See below for STORM imaging of CB_1_. Confocal z-stacks containing the entire recovered arborization of the filled neurons were collected on a C2 confocal system (Nikon). For the visualization of representative cells, two cells were manually reconstructed in Neurolucida software (MBF Bioscience). For the analysis of dendritic morphology, image stacks were processed with ImageJ software (NIH), using identical parameters across all images. All visible branches of the dendrites were then manually reconstructed in 3D using Neuronstudio software (Rodriguez et al. 2006). Sholl analysis was performed in Neuronstudio to measure the length of dendrites traversing a concentric series of spherical shells with 1 μm increments in radius. Then, the measured dendritic lengths were averaged to form bins with 25 μm increments. Cells were pooled by treatment group, and the differences in length values in each bin were tested using a two-sample Kolmogorov–Smirnov test. For the quantitative analysis of axonal morphology, the bouton distribution index (BDI) was calculated for each cell as described previously (Dudok et al. 2015). Briefly, the positions of axonal varicosities were recorded as a distance from the borders of the pyramidal layer. The laminar distribution of the varicosities was then visualized as the histogram of relative distances (where 0 is the center and 1 is the thickness of the pyramidal layer), and the BDI was calculated from the descriptive statistics of the distribution. Cells with BDI >1 were included in the study as perisomatically targeting interneurons, while all other cells were excluded.

### Stochastic optical reconstruction microscopy (STORM) super-resolution imaging

After imaging the developed cells as described above, immunostaining, correlated confocal and STORM microcopy, and image analysis were carried out according to the previously described protocol (Barna et al. 2016). Slices containing filled cells were embedded in agarose and resectioned to 20 micron thickness. Immunostaining was performed using a previously validated primary raised in Guinea pig against the C terminus of CB_1_ (1:1000 in TBS) (Fukudome et al. 2004; Dudok et al. 2015) and Alexa 647-conjugated secondary antibodies (Jackson ImmunoResearch, 2 µg/mL in TBS). Before imaging, sections were covered with Dulbecco’s phosphate buffered saline containing 5% (wt/vol) glucose, 0.1 M 2-Mercaptoethylamine (Sigma), 1 mg/mL glucose oxidase (Sigma), and 1500 U/mL catalase (Sigma). Images were collected using Nikon C2 confocal microscope. Then, astigmatic 3D-STORM (Huang et al. 2007) images of the immunostaining were recorded in continuous dSTORM mode (Heilemann et al. 2008) for 5000 frames at 31 Hz. Confocal image stacks were deconvolved using Huygens software (SVI). STORM images were processed for peak detection using the N-STORM module in NIS-Elements software (Nikon). The average lateral localization precision was 9.6 ± 0.9 nm. Correlated pairs of confocal images and molecule lists were loaded in VividSTORM software (Barna et al. 2016), aligned, and regions of interests (ROI) containing labeled axon terminals were defined using unbiased active contour algorithm. The perimeter of the ROI, as well as the STORM NLP within the ROI, were determined for each axon terminal. Density values were calculated as NLP over perimeter. As the Kruskal–Wallis test detected significant difference between the cells within groups, cells were not pooled but the average values were calculated from 12 ± 3 axon terminals per cell. These values were then compared between treatment groups using Mann–Whitney *U* test. The internalization of CB_1_ was assessed by calculating the internalization index as reported earlier, where this measure could readily detect increased CB_1_ internalization upon in vivo THC exposure (Dudok et al. 2015). All images were recorded, processed and analyzed with identical settings.

### Liquid chromatography/mass spectrometry analyses

Whole hippocampi were collected from C57BL/6J mice 7 weeks after irradiation, quickly frozen on dry ice, and stored at −70 °C until the lipid analyses. Amounts of 2-AG in the dissected hippocampi were determined as described (Astarita and Piomelli 2009). Briefly, frozen tissue samples were homogenized in cold methanol (1 ml) containing 2-arachidonyl glycerol-d_8_ (2-AG-d_8_; Cayman Chemical, Ann Arbor, MI) as an internal standard. Protein concentration was determined in the homogenate to normalize samples using the bicinchinonic acid (BCA) protein assay (Pierce, Rockford, IL, USA). Lipids were extracted by adding chloroform and water (2:1) and fractionated through open-bed silica gel columns (60-Å 230–400 Mesh ASTM; Whatman, Clifton, NJ) by elution with 1 ml of chloroform/methanol (9:1). Eluates were dried under N_2_, and reconstituted in chloroform/methanol (1:3 μl).

We used an Agilent 1100-LC system coupled to a 1946A-MS detector equipped with an electrospray ionization interface (Agilent Technologies, Inc., Palo Alto, CA). Lipids were separated on a reversed-phase XDB Eclipse C18 column (50 × 4.6 mm i.d., 1.8 μm, Zorbax, Agilent Technologies). They were eluted with a gradient of methanol in water (from 85 to 90% methanol in 2.0 min and 90–100% in 3.0 min) at a flow rate of 1.5 ml/min. Column temperature was kept at 40 °C. Mass spectrometry detection was in the positive ionization mode, capillary voltage was set at 3 kV and fragmentor voltage was 120 V. N_2_ was used as drying gas at a flow rate of 13 l/min and a temperature of 350 °C. Nebulizer pressure was set at 60 PSI. For quantification purposes, we monitored the sodium adducts of the molecular ions [M+Na]^+^ in the selected ion-monitoring mode, using 2-AG-d_8_ (mass-to-charge ratio for 2-AG-d_8_: *m*/*z* = 409) as internal standards.

### Statistics

Paired or unpaired two-tailed Student’s *t* tests were used when the data showed a normal distribution; otherwise Wilcoxon’s signed rank (paired data) or Mann–Whitney tests (unpaired data). Pearson’s Chi-squared tests were used for the connection probability. Other statistical tests are noted in the text. Data are presented as mean ± SEM. A *p* value <0.05 was considered significant. Statistical analyses were performed using Origin Pro 2015 (OriginLab Corporation, Northampton, MA, USA), STATISTICA 10 (Dell Statistica, Tulsa, OK, USA), and GraphPad QuickCalcs (GraphPad Software, La Jolla, CA, USA).

## Results

### Irradiation does not alter morphological or intrinsic properties of CB_1_BCs

Our past work using identical irradiation paradigms demonstrated that whole body proton irradiation at the space relevant dose of 0.5 Gy (Cucinotta et al. 2014; Nelson 2016) impairs cortical and hippocampus-dependent learning and memory (Parihar et al. 2015b). To determine whether similar exposures caused long-term alterations in inhibitory synaptic transmission, paired patch-clamp recordings from identified interneurons and PCs in the CA1 region of the hippocampus were performed in acute slices from irradiated and control mice two months after irradiation.

**Fig. 1:**
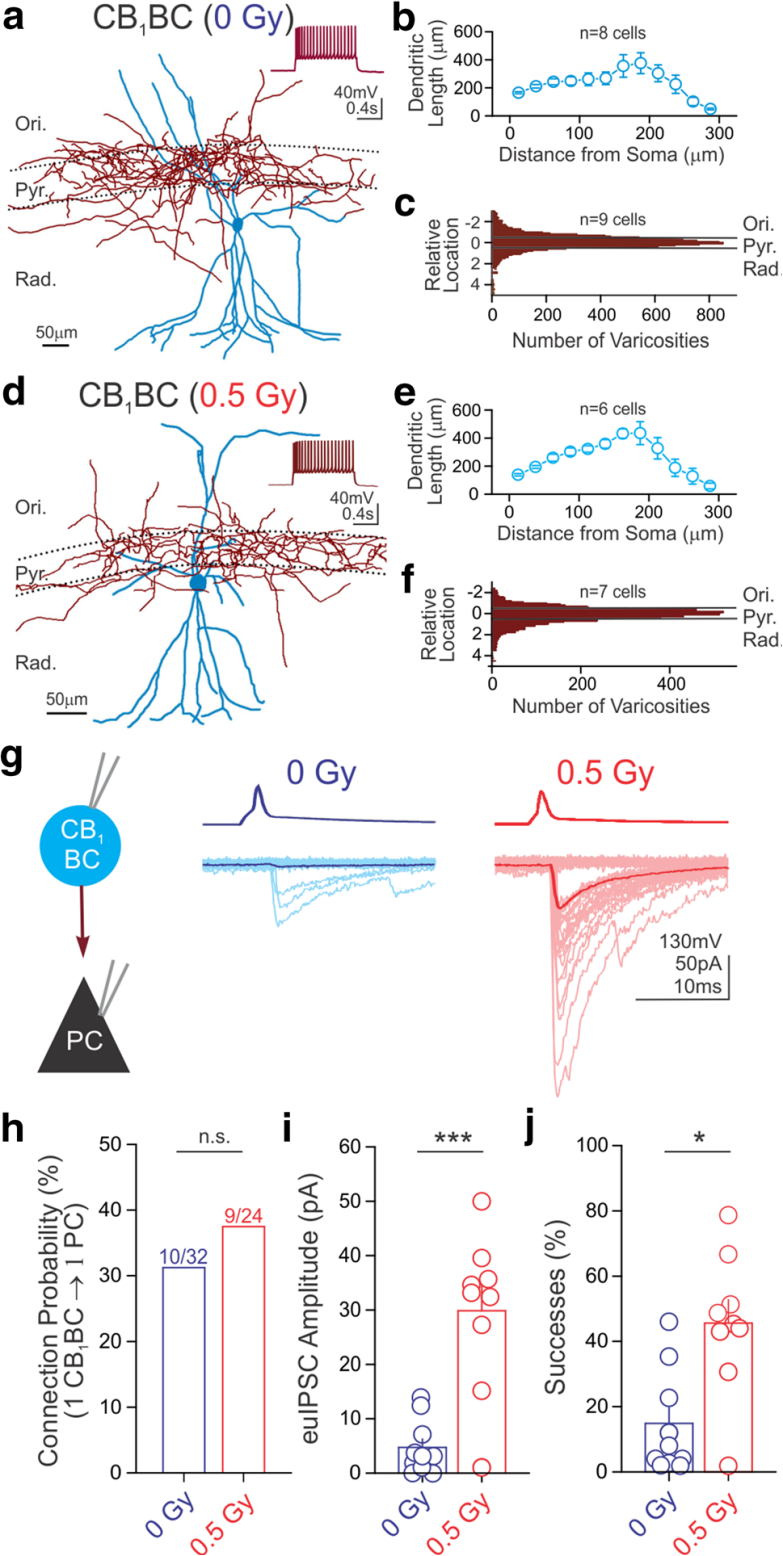
Proton irradiation increases CB_1_-sensitive GABA release. Representative tracings of CB_1_BCs from control (**a**) and irradiated (**d**) mice (*blue* dendritic tree and soma; *red* axonal arbor). *Insets* demonstrate the characteristic spike frequency adaptation in response to a depolarizing current step (+200 pA, from −65 mV). Sholl analysis of dendritic trees revealed similar mean dendritic length between control (**b**) and irradiated (**e**) mice (length per 25 μm bin is shown as a function of distance from the cell body; two-sample Kolmogorov–Smirnov test, *p* > 0.1 in each bin of graded radius at 25 μm steps). The distribution of axonal varicosities was taken as the distance of each varicosity from the center of the stratum pyramidale (which is denoted as 0 on the *y axis*), normalized to the thickness of the layer. Pooled distributions of CB_1_BC axonal arbors were similar for control (**c**) and irradiated (**f**) mice. **g** Representative traces of paired recordings from presynaptic CB_1_BCs (*top*, AP) and postsynaptic PCs (*bottom*, IPSCs) from a control and an irradiated mouse. Fifty consecutive traces (*light lines*) and their averages (*dark lines*) are presented here and in all subsequent figures. Summary data plots demonstrate that irradiation did not affect CB_1_BC to PC cell connection probability [**h**; *numbers above bars* = (# connected)/(# tested)], but did result in significant increases of euIPSC amplitudes (**i**) and successes of postsynaptic events (**j**). *Ori* stratum oriens, *Pyr* stratum pyramidale, *Rad* stratum radiatum. For all figures: *p < 0.05; **p <0.01; ***p < 0.005; *ns* not significant

Because recent studies have shown that high- and low-dose irradiation paradigms can compromise dendritic structures (Parihar and Limoli 2013; Allen et al. 2015) and intrinsic properties (Sokolova et al. 2015) of hippocampal principal neurons, we carried out a detailed analysis of the neuronal morphology and intrinsic properties of CB_1_BCs from control and proton-irradiated animals. Every CB_1_BC included in this study was rigorously identified based on the immunopositivity of its axon terminals for CB_1_ (Fig S1) and the predominant localization of its axons within the striatum pyramidale (Fig. 1a, c, d, f). Detailed quantitative analysis showed no differences between CB_1_BCs from controls and irradiated mice in dendritic morphology (Fig. 1), including the number of segments, branch points and length (Fig S1B**-**D). Axonal morphology was also unchanged (Fig. 1), including the bouton distribution index (a measure of axons in the pyramidal cell layer compared to the dendritic layers; Dudok et al. 2015), bouton density and area of boutons (Fig S1E**-**G). Similarly, proton irradiation did not change passive or active electrophysiological properties of CB_1_BCs (Fig S2). These experiments showed that the morphological and intrinsic properties of CB_1_BCs remained unaltered by low-dose proton irradiation, in contrast to what has been shown in excitatory hippocampal cells (Parihar et al. 2014; Sokolova et al. 2015).

### Low-dose proton irradiation increases GABA release from CB_1_BCs

Next, we examined possible alterations in proton-induced persistent plasticity in GABAergic neurotransmission using paired patch-clamp recordings from identified presynaptic CB_1_BCs and postsynaptic CA1 PCs. Because of heterogeneity amongst CA1 PCs (Lee et al. 2014), we selectively recorded from PCs within the superficial sublayer. Irradiation did not affect the GABAergic connection probability (proportion of potential pairs in which there was successful GABAergic transmission) between CB_1_BCs and PCs (Fig. 1h; connected/tested = 10/32, 0 Gy; 9/24, 0.5 Gy). These data were in agreement with a lack of alterations in axonal arborization of CB_1_BCs discussed above. Nevertheless, irradiation led to a robust, 6.3-fold increase in the CB_1_BC-evoked IPSCs in PCs (Fig. 1g, i; IPSC amplitudes, including both successes and failures are “effective unitary IPSCs” or euIPSCs: 0 Gy, 4.7 ± 1.5 pA, *n* = 10; 0.5 Gy, 29.9 ± 4.8 pA, *n* = 9; *p* < 0.005). In addition, there was a proton-induced increase in the percentage of successful events evoked by APs in CB_1_BCs (Fig. 1j, % success: 0 Gy, 14.9 ± 4.8%, *n* = 10; 0.5 Gy, 45.6 ± 7.1%, *n* = 9; *p* < 0.05), suggesting that irradiation resulted in an increased probability of AP-dependent GABA release. Indeed, the quantal content of the unitary inhibitory responses increased (as determined by changes in 1/CV^2^) in the irradiated group (1/CV^2^: 0.20 ± 0.07 in controls; 0.64 ± 0.11 in the irradiated group; *p* < 0.05; *n* = 10 and 9 pairs). These data show a marked, long-term increase in GABAergic synaptic transmission after irradiation.

### Irradiation causes a reduction in the tonic cannabinoid-mediated inhibition of GABA release

**Fig. 2:**
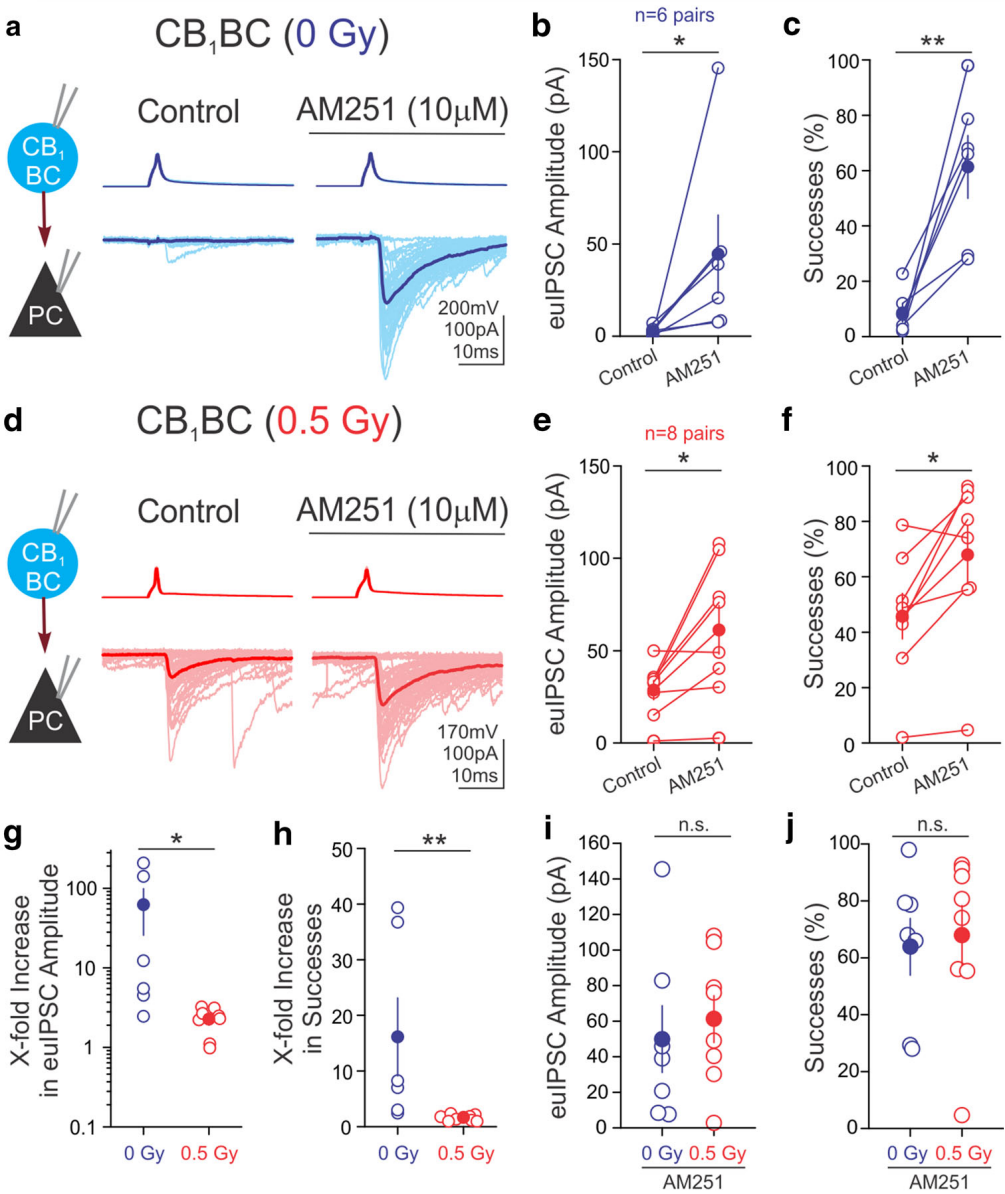
Reduced tonic cannabinoid signaling by irradiation. Representative traces of paired recordings from presynaptic CB_1_BCs (*top*, AP) and postsynaptic PCs (*bottom*, IPSCs) in control ACSF (Control) and during application of AM251 from control group (**a**) and irradiated group (**d**). Summary of the effects of AM251 on euIPSCs in control (**b**) and irradiated (**e**) groups. Percent of presynaptic APs resulting in successful IPSCs (successes) in the postsynaptic cell in control (**c**) and irradiated (**f**) groups. Application of AM251 causes more of an increase in euIPSCs (**g**) and successes (**h**) in controls than after irradiation. After application of AM251, the amplitude of euIPSCs (**i**) and successes (**j**) are not different between groups (*n* = 7 pairs, 0 Gy; *n* = 8 pairs, 0.5 Gy)

CB_1_s are the most abundant G-protein-coupled receptors in the brain, selectively localized on the axon terminals of CCK-containing CB_1_BCs (Katona et al. 1999; Mackie 2005; Soltesz et al. 2015). GABA release from CB_1_BCs is known to be under strong inhibition caused by CB_1_-dependent tonic cannabinoid signaling (Neu et al. 2007; Lee et al. 2010, 2015; Dudok et al. 2015). Therefore, we tested the hypothesis that the irradiation-induced increase in euIPSCs evoked by CB_1_BCs in PCs (Fig. 1g**–**j) was due to a loss of the cannabinoid-mediated tonic control of GABA release. We found that the CB_1_ antagonist/inverse agonist AM251 (10 μM; in perfusate) increased the euIPSC amplitudes and the probability of successful release in CB_1_BC-PC pairs from control mice (Fig. 2a**–**c; ACSF: 2.8 ± 1.0 pA, 8.6 ± 3.2%, *n* = 10; AM251, 44.5 ± 21.2 pA, 61.3 ± 11.3%, *n* = 6; *p* < 0.05). Although AM251 also increased the euIPSC amplitudes and probability of success in irradiated mice (Fig. 2d**–**f; ACSF: 28.7 ± 5.2 pA, 45.8 ± 8.1%, *n* = 9; AM251: 61.2 ± 13.1 pA, 67.9 ± 10.4%; *n* = 8; *p* < 0.05), these effects were dramatically lower after irradiation (Fig. 2g, h; AM251-induced increases in euIPSCs: 0 Gy: 62.1 ± 36.4 fold; 0.5 Gy: 2.3 ± 0.3 fold; AM251-induced increases in successful release: 0 Gy: 16.1 ± 7.0 fold; 0.5 Gy: 1.7 ± 0.2 fold; *n* = 6 and 8, *p* < 0.05). Importantly, the proton-induced increases in euIPSCs in the CB_1_BC-PC pairs (Fig. 1g**–**j) could be attributed to a decreased cannabinoid control of GABA release, because there were no differences in euIPSCs between control and irradiated mice in the presence of AM251 (Fig. 2i, j; euIPSCs: 0 Gy: 50.1 ± 18.7 pA, *n* = 7; 0.5 Gy: 61.2 ± 13.1 pA, *n* = 8, p = 0.63; successful probability of release: 0 Gy: 63.9 ± 9.9%, *n* = 7; 0.5 Gy: 67.9 ± 10.4%, *n* = 8, *p* = 0.68). These data demonstrated that space-relevant doses of proton irradiation caused persistent, long-term increases in GABA release from CB_1_BC interneurons, and that the potentiated GABA release was due to a less effective tonic, inhibitory control of GABA release by CB_1_s.

### Irradiation does not alter CB_1_ numbers, but depresses 2-AG levels

**Fig. 3:**
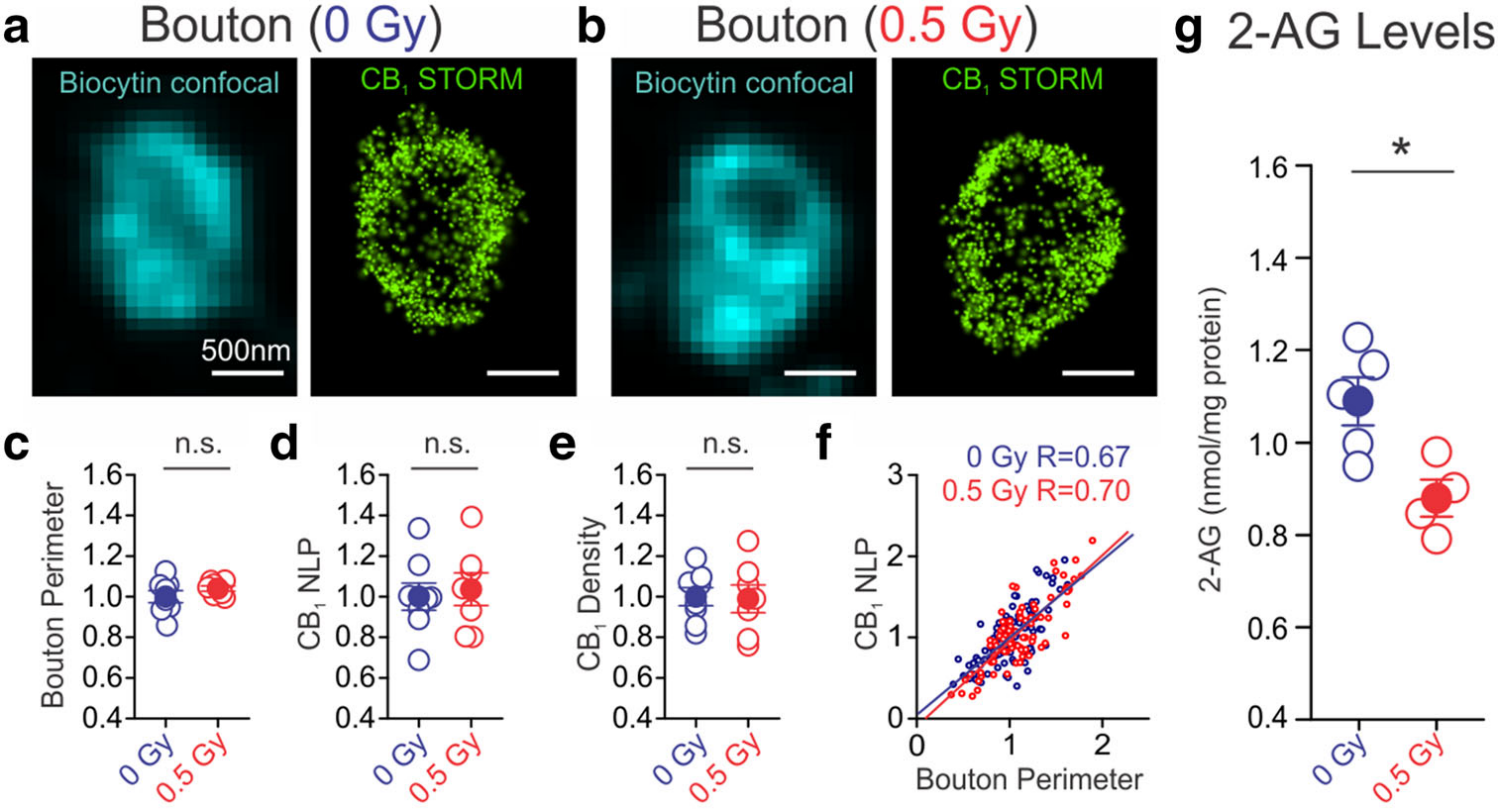
Irradiation reduced 2-arachidonoylglycerol (2-AG) levels, but not CB_1_ content. **a**, **b** Super-resolution images of CB_1_s of axon terminals of CB_1_BCs were obtained with correlated confocal microscopy and STORM microscopy. Localization points (*green dots*) in the STORM images represent the position of CB_1_s in the axon terminals. There are no changes in CB_1_ expression in CB_1_BCs as measured by bouton perimeters (**c**), CB_1_ NLP (**d**), and CB_1_ density (NLP/bouton perimeter) (**e**). *Open circles* represent mean values of each cell from 12 ± 3 boutons per cell normalized to the mean of control cells. *Blue* or *red filled circles* label averages of control and irradiated groups. **f** There were moderately strong correlations between CB_1_ NLP and bouton perimeter in both groups of boutons (*n* = 107 and 78 boutons from control and irradiated mice, respectively). **g** Irradiation led to a decrease in 2-AG levels (*n* = 5 control, *n* = 4 irradiated)

Tonic CB_1_-dependent inhibition of GABA release is influenced by endocannabinoid-mediated (ligand-driven) steady-state baseline activation of CB_1_, as well as by constitutive (ligand-free) CB_1_ activity (Lee et al. 2015). Therefore, one possible explanation for the decreased tonic cannabinoid signaling after proton exposure (Fig. 2) is a reduction in CB_1_ levels. To test this possibility, correlated confocal and STORM super-resolution imaging (Dudok et al. 2015; Barna et al. 2016) was used to analyze the axon terminals of biocytin-filled CB_1_BCs (control: *n* = 107 boutons, 8 CB_1_BCs, 5 mice; irradiated: *n* = 78 boutons, 7 CB_1_BCs, 3 mice). Irradiation did not cause alterations in axon terminal size, quantified as the perimeter of optical cross-sections of CB_1_-expressing boutons (Fig. 3a**–**c). There were no changes in the number of CB_1_ localization points (NLP; control: *n* = 1322.1 ± 234.1 NLP/bouton, 8 CB_1_BCs; irradiated: *n* = 1370.2. ± 260.3 NLP/bouton, 7 CB_1_BCs; Fig. 3d). As a result, the specific CB_1_ content of axon terminals (NLP over perimeter) also remained unchanged (Fig. 3e). Note that bouton size and CB_1_ content showed similar correlations in control and irradiated groups (Fig. 3f; Spearman’s rank order correlation, *p* < 0.001 in both groups). In addition, there was no change in the internalization of CB_1_ receptors after irradiation, as revealed by measurements of the distance of individual localization points from the outline of the axon terminals (*n* = 107 and 78 boutons from control and treated cells, respectively).

The endocannabinoid 2-AG is known to be able to mediate tonic cannabinoid signaling at CB_1_-expressing synapses (Hashimotodani et al. 2007; Anderson et al. 2015; Lee et al. 2015). To determine if 2-AG levels underwent long-term proton-induced alterations in the hippocampus, we measured its concentrations in whole hippocampi by liquid chromatography/mass spectrometry. We found that irradiation led to decreased levels of 2-AG (Fig. 3g; 0 Gy: 1.09 ± 0.05 nmol/mg protein, *n* = 5; 0.5 Gy: 0.88 ± 0.04 nmol/mg protein, *n* = 4; *p* < 0.05). Therefore, these imaging and biochemical data showed the robust post-irradiation increases in CB_1_BC-evoked IPSCs were not due to a loss of cannabinoid-mediated inhibition of GABA release resulting from a down-regulation of CB_1_ numbers on the CB_1_BC axon terminals. They were, however, accompanied by reduced 2-AG levels.

### PVIN-mediated inhibition remains unaltered after irradiation

**Fig. 4:**
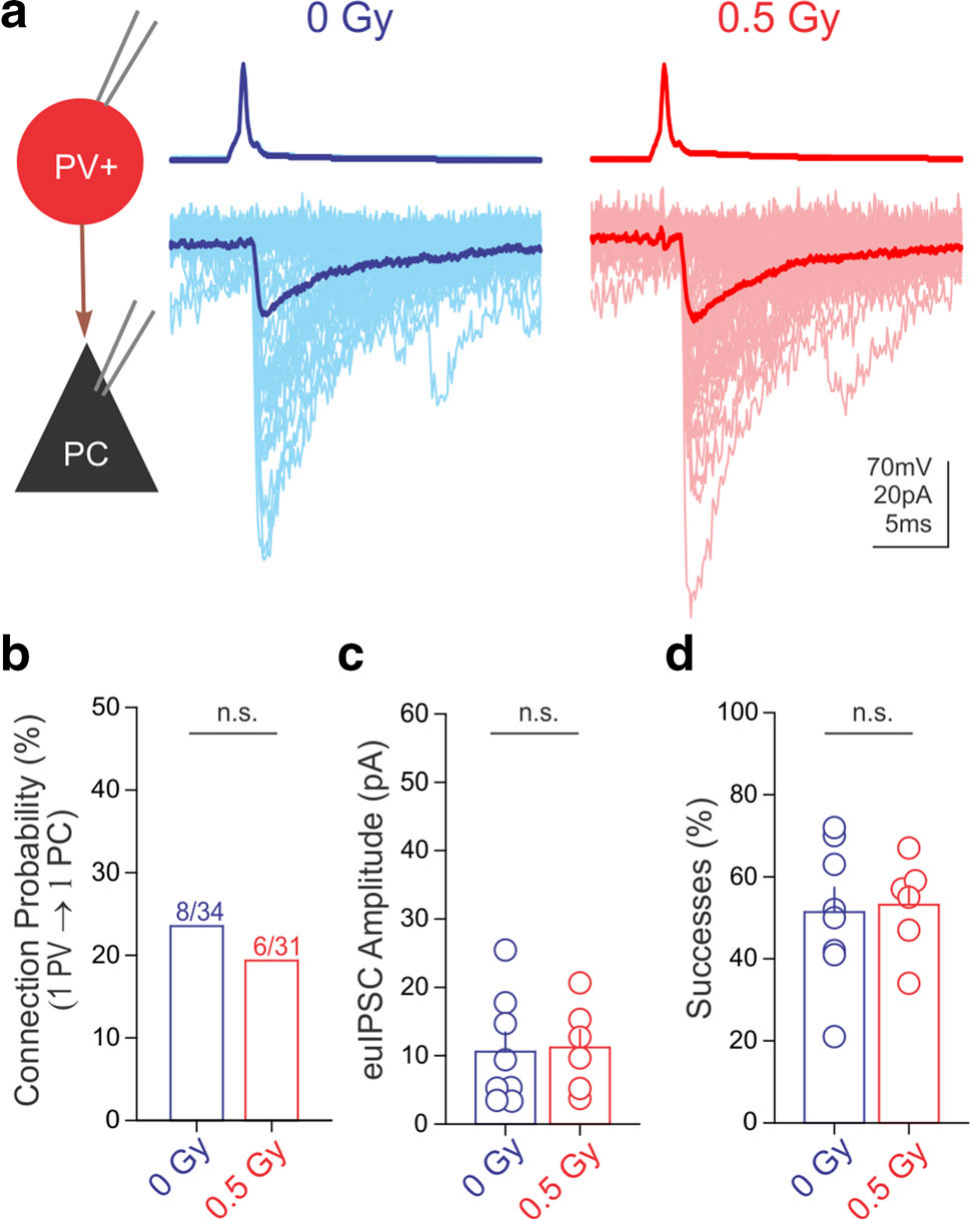
Irradiation did not alter GABA release from PVINs. **a** Representative traces of paired recordings from presynaptic PVINs (*top*, AP) and postsynaptic PCs (*bottom*, IPSCs) from a control and an irradiated mouse. Summary data plots demonstrate that irradiation did not affect PVIN to PC connection probability (**b**), euIPSC amplitudes (**c**), or successes of postsynaptic events (**d**)

Next, we examined whether PVIN-mediated GABA release was also enhanced by irradiation. These experiments provided an important set of controls, since PVINs do not express CB_1_s but are thought to be indispensable for network activity associated with hippocampus-dependent cognition (Katona et al. 1999; Fuchs et al. 2007; Sohal et al. 2009; Stark et al. 2014; Hu et al. 2014). Similar to what was observed for CB_1_BCs, irradiation did not alter any of the intrinsic properties analyzed (Fig S3), and the paired recordings also revealed no differences in the GABAergic connection probability between presynaptic PVINs and PCs (Fig. 4a, b: connected/tested 0 Gy = 8/34; 0.5 Gy = 6/31). However, in stark contrast to the robust potentiation of unitary IPSCs between CB_1_BCs and PCs, irradiation did not change the IPSC amplitudes or the probability of successful release in PVIN-PC pairs (Fig. 4c, d; euIPSC amplitudes: control: 10.6 ± 2.8 pA, *n* = 8; irradiated: 11.2 ± 2.6 pA, *n* = 6, *p* > 0.5; % successful release: control: 51.4 ± 6.0%, *n* = 8; irradiated: 53.2 ± 4.7%, *n* = 6, *p* > 0.5). Thus, data derived from two distinct interneuronal classes demonstrated that irradiation caused highly cell type-specific, persistent alterations in GABAergic synaptic transmission in the hippocampus.

### Irradiation selectively increases the excitatory connection probability between PCs and PVINs

**Fig. 5:**
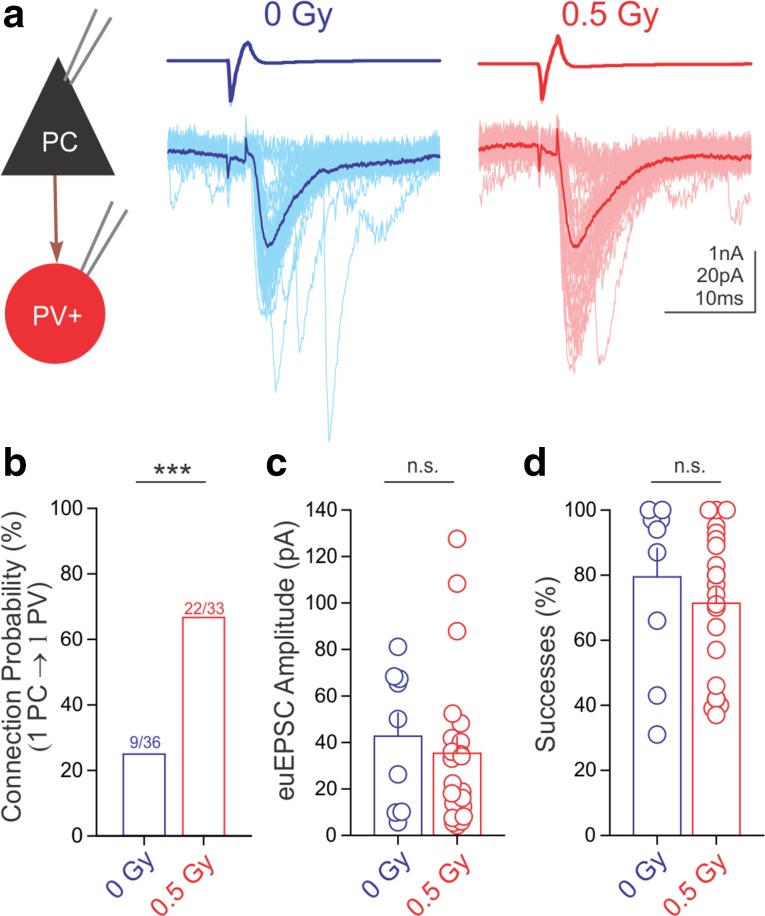
Irradiation selectively increased the connection probability between CA1 PCs and PVINs. **a** Representative traces of paired recordings from presynaptic PCs (*top*, action currents) and postsynaptic PVINs (*bottom*, EPSCs) from a control and an irradiated mouse. Summary data plots demonstrate that irradiation led to an increase in PC to PVIN connection probability (**b**), but did not affect the amplitude of euEPSCs (**c**) or the successes of postsynaptic events (**d**)

Thus far, we have focused on the GABAergic transmission from the CB_1_BCs and PVINs to the CA1 PCs. These studies do not, however, address whether proton irradiation alters the glutamatergic transmission from CA1 PCs to either type of interneuron. To investigate this, the PC-PVIN pairs were analyzed in the reverse order than in the experiments described above. Paired recordings revealed large radiation-induced increases in excitatory connection probability between presynaptic PCs and postsynaptic PVINs (Fig. 5a, b; connected/tested = 9/36 (control), 22/33(irradiated); *p* < 0.005). Therefore, excitatory glutamatergic connections also undergo long-term alterations following proton irradiation. However, proton irradiation did not affect the effective unitary excitatory postsynaptic (euEPSC) amplitudes or the probability of successful release in PC to PVIN pairs (Fig. 5c, d; EPSC amplitudes: 0 Gy: 42.7 ± 9.9 pA, *n* = 9; 0.5 Gy: 35.3 ± 7.1 pA, *n* = 22, *p* > 0.5; probability of success: control: 79.4 ± 8.8%, *n* = 9; irradiated: 71.4 ± 4.7%, *n* = 22, *p* > 0.5).

Since CA1 PCs very rarely innervate CB_1_BCs in control mice (Lee et al. 2014), we investigated whether irradiation also increases the excitatory connections between CA1 PCs and CB_1_BCs. Unlike what we observed for PC-PVIN connections, paired recordings revealed no increases in the excitatory connection probability between the PCs and the CB_1_BCs [connected/tested: 0/23(control), 0/13(irradiated)]. These experiments demonstrated that proton irradiation caused highly cell type-selective alterations in both the excitatory and inhibitory synaptic microcircuits within the CA1 region of the hippocampus.

## Discussion

In the present study, we systemically examined the effects of space-relevant, low-dose proton irradiation on local synaptic circuits within the hippocampus, a brain area centrally important for some of the cognitive tasks affected by radiation exposure (Britten et al. 2012, 2014; Bellone et al. 2015). The key findings are: (1) Proton irradiation resulted in a large, persistent potentiation of action potential-dependent GABA release from CB_1_BCs onto CA1 PCs, without alterations in the axonal or dendritic morphology or intrinsic excitability of these interneurons; (2) The mechanism of the radiation-induced increase in GABA release from CB_1_BCs was a significant decrease in the CB_1_-mediated control of GABA release, associated with depressed 2-AG levels; (3) The effects of irradiation on GABA release were cell type-specific, since proton exposure did not alter GABA release from PVINs; (4) Local excitatory circuits were also modulated by radiation, as indicated by a proton-induced enhancement of the connection probability between PCs and PVINs, without significant increases in the PC to CB_1_BC connections. Therefore, we have demonstrated that energetic solar particles selectively alter local GABAergic inhibition and glutamatergic excitation in the hippocampus.

### Cellular-synaptic bases of persistent cognitive impairments associated with radiation exposures relevant to interplanetary travel

With recent advances in space exploration, the possibility of human interplanetary travel no longer seems like science fiction. Before sending humans on such long-term voyages in space, the immediate and long-term health risks caused by exposure to space radiation fields must be carefully evaluated. Decades of clinical experience in the management of brain tumors have revealed the adverse effects of cranial irradiation on cognition (Meyers 2000; Butler et al. 2006). While clinical irradiation scenarios are clearly distinct (i.e., in terms of radiation dose and type) from those in space, significant recent work using rodent models has now corroborated that very low doses of charged particles can disrupt cognition using a variety of behavioral tasks (Britten et al. 2012; Lonart et al. 2012; Parihar et al. 2015a).

In addition, these decreases in cognitive performance were associated with structural changes in dendrites and alterations in key synaptic proteins (Parihar and Limoli 2013; Parihar et al. 2014, 2015a; Allen et al. 2015; Chmielewski et al. 2016). However, our understanding of how charged particles may impact specific excitatory and inhibitory circuits in the brain has been limited. In this paper, we studied the effects of the space relevant dose of 0.5 Gy on key hippocampal microcircuits. We chose to focus on the effect of protons, given that they constitute the vast majority of charged particles in space (Cucinotta et al. 2014; Nelson 2016). We carried out our experiments in the hippocampus, because it plays crucial roles in cognitive tasks that include memory consolidation and spatial navigation. Hippocampal perisomatic inhibition is mediated by CB_1_BCs and the numerically dominant PV basket cells within the PVIN class. These cells are specialized to form multiple synaptic contacts on the somata and proximal dendrites of hundreds of postsynaptic PCs. Because of the proximity of their inhibitory output synapses to the AP initiation site located on the initial segment of PCs, perisomatically projecting interneurons are in a strategic position to control hippocampal network output. Importantly, there is a strict division of labor between CB_1_BCs and PVINs. Hippocampal PVINs receive large amounts of excitatory inputs, fire fast and non-accommodating APs, have fast membrane time constants, and release GABA in synchrony with presynaptic APs (Armstrong and Soltesz 2012). Thus, PVINs can function as the precisely timed inhibitory elements of the hippocampal circuit and play key roles as time-keepers in the generation of distinct behaviorally relevant network rhythms, including theta and gamma oscillations and sharp wave ripples. In contrast, CB_1_BCs are the more modifiable elements of perisomatic inhibitory control (Armstrong and Soltesz 2012), since these cells express an especially large variety of receptors for various neuromodulators, including endocannabinoids. Pathological alterations in PVIN and CB_1_BC properties and functions have been reported in a variety of neurological disorders, including epilepsy, schizophrenia, autism, and Huntington disease (Chen et al. 2003; Curley and Lewis 2012; Dvorzhak et al. 2013; Földy et al. 2013).

Here we have shown that the effect of proton irradiation has long-lasting, highly specific effects on hippocampal perisomatic inhibitory microcircuits, and that the radiation-induced plasticity involves not only the interneuronal inputs from CB_1_PCs to PCs but also the excitatory innervation of PVINs by local CA1 PCs. The specificity of these effects indicate that proton irradiation does not indiscriminately affect the synaptic properties of neuronal circuits, in spite of the fact that the effect size was large, specifically, a more than sixfold increase in GABA release from CB_1_BCs without any alteration in the release properties from PVINs. The specificity and magnitude of these persistent alterations in perisomatic inhibitory circuits are consistent with the marked alterations in cognitive performance after space-relevant doses of radiation (Lonart et al. 2012; Bellone et al. 2015). Indeed, given the reported roles of CB_1_BCs in a variety of circuit functions, including the integration of synaptic inputs from a variety of local and long-distance sources, as well as in the modulation of the input–output gains of CA1 PCs during network activity and the expression of input-timing depending plasticity, the robust increase in GABA release from CB_1_BCs after proton irradiation likely affects the assessment of the saliency of inputs arriving at the hippocampus from the entorhinal cortex (Armstrong and Soltesz 2012; Basu et al. 2013). In turn, the resulting lack of proper filtering of incoming salient information about the environment is expected to lead to aberrant memory consolidation. Similarly, PVINs are thought to be involved in the generation of oscillations in the hippocampal network, particularly the high frequency gamma oscillations, through their fast-spiking properties and their strong connectivity with PCs (Fuchs et al. 2007; Sohal et al. 2009; Holderith et al. 2011; Buzsáki and Wang 2012; Hu et al. 2014). Therefore, alterations to the excitatory inputs of PV cells after proton irradiation likely compromise the precise spike timing necessary for the generation of gamma oscillations, with downstream effects on associated cognitive functions, such as spatial memory, attention, and cognitive flexibility (Isaacson and Scanziani 2011; Buzsáki et al. 2012; Cho et al. 2015; Kim et al. 2016). These results indicate that irradiation-related pathological alterations of either CB_1_BCs or PVINs could result in deficits in hippocampus-dependent cognitive function.

Our findings indicating selective radiation-induced alterations to inhibitory and excitatory synapses are in overall agreement with previous reports of both hypo- and hyperexcitable modifications in hippocampal circuits after space-relevant doses of radiation. For example, the present data showing increased GABA release from CB_1_BCs and enhanced excitatory innervation of PVINs suggest an augmentation of perisomatic inhibition of PCs, in agreement with findings indicating hyperpolarized resting membrane potential and decreased input resistance of PCs after proton irradiation (Sokolova et al. 2015). However, perisomatic inhibition can modulate hippocampal excitability in complex ways (Armstrong and Soltesz 2012). For example, increased inhibitory inputs can cause paradoxical rebound spiking and increases in synchronized discharges in hippocampal circuits (Cobb et al.^1995^; Chen et al. 2001). Space-relevant irradiation using low doses of protons or high-energy charged particles (e.g., ^28^Si and ^56^Fe) generally resulted in an overall increase in hippocampal excitability as assessed by field EPSPs in the CA1 and the dentate gyrus (Vlkolinský et al. 2007; Marty et al. 2014; Rudobeck et al. 2014; Bellone et al. 2015). Similarly, reports indicating increases in postsynaptic density protein (PSD-95) expression, persistent sodium currents, and excitatory synaptic transmission are also consistent with hippocampal hyperexcitability caused by space-relevant irradiation (Parihar et al. 2014; Sokolova et al. 2015). Computational modeling—taking into account some of these proton-irradiation-induced complex alterations in cellular and synaptic excitability—suggested that a perturbation in behaviorally relevant theta-frequency oscillations may take place in hippocampal networks (Sokolova et al. 2015) that could partially underlie radiation-induced disturbances in cognitive performance.

### Perturbation of the endocannabinoid system and neurological dysfunction

Our experiments identified that loss of CB_1_-mediated tonic inhibitory control was a major factor underlying the marked upregulation of GABA release from CB_1_BCs after proton irradiation. In the hippocampus, CB_1_s are highly expressed on axon terminals of specific subtypes of GABAergic interneurons (e.g., CB_1_BCs), as well as on subsets of excitatory terminals (Katona et al. 1999; Mackie 2005; Soltesz et al. 2015). The activity of presynaptic CB_1_s exerts robust inhibition of GABA release (Neu et al. 2007; Hashimotodani et al. 2007; Lee et al. 2010; Kim and Alger 2010; Lee et al. 2015). Evidence is mounting that perturbations of tonic cannabinoid signaling occur in a variety of neurologic disorders including epilepsy, Fragile X syndrome, autism, schizophrenia and chronic ethanol exposure (Chen et al. 2003; Maccarrone et al. 2010; Curley and Lewis 2012; Dvorzhak et al. 2013; Földy et al. 2013; Tang and Alger 2015; Varodayan et al. 2016). Additionally, the knockout of β-neurexins, important cell adhesion molecules, leads to decreased tonic endocannabinoid signaling and results in impairment of contextual memory (Anderson et al. 2015), and genetic deletion of CB_1_s from GABAergic interneurons results in hippocampus-dependent spatial memory impairments (Albayram et al. 2016). Therefore, our results indicating that proton irradiation leads to a large, long-term decrease in CB_1_-mediated tonic inhibition of GABA release from CB_1_BCs to PCs, accompanied by decreased levels of 2-AG, are consistent with the sensitivity of the endocannabinoid signaling system to a variety of perturbations, with significant consequences for circuit performance. Basal levels of 2-AG are tightly controlled by the activities of both diacylglycerol lipase-α (2-AG synthetic enzyme), which is highly localized on dendritic spines of PCs, and monoacylglycerol lipase (2-AG degrading enzyme), which is localized on excitatory and inhibitory presynaptic terminals as well as astrocytes (Soltesz et al. 2015; Lee et al. 2015). Therefore, the decreased levels of 2-AG following proton irradiation may be due to downregulated diacylglycerol lipase-α and/or upregulated monoacylglycerol lipase, and future investigations will be conducted to discriminate between these and potentially other possibilities.

In summary, our results demonstrate that space relevant doses of proton irradiation causes persistent, large and highly specific alterations to perisomatic inhibitory circuits. Given the crucial roles that the perisomatically projecting interneurons play in hippocampus-dependent cognitive tasks, these changes are likely to be mechanistically linked to the cognitive effects found after radiation exposure in a variety of settings including space travel. The specific nature of the radiation-induced changes in perisomatic circuits may also present future opportunities for designing novel therapeutic avenues targeting the versatile cannabinoid signaling system (Soltesz et al. 2015), as well as other molecular pathways that may be involved.

## Electronic supplementary material

Below is the link to the electronic supplementary material.

Supplementary material 1 (DOCX 1415 kb)
