# Supplementary material for: Neurophysiology of space travel: energetic solar particles cause cell type-specific plasticity of neurotransmission
Source: Brain Struct Funct. 2016 Nov 30;222(5):2345–57. doi: 10.1007/s00429-016-1345-3 (PMC5504243; doi:10.1007/s00429-016-1345-3)
Supplement: Supplementary file 1 — Supplementary material 1 (DOCX 1415 kb) [file 429_2016_1345_MOESM1_ESM.docx]

**Supplementary Materials**

**Neurophysiology of space travel: Energetic solar particles cause cell type-specific plasticity of neurotransmission**

Sang-Hun Lee, Barna Dudok, Vipan K. Parihar, Kwang-Mook Jung, Miklós Zöldi, Young-Jin Kang, Mattia Maroso, Allyson L. Alexander, Gregory A. Nelson, Daniele Piomelli, István Katona, Charles L. Limoli, Ivan Soltesz

**
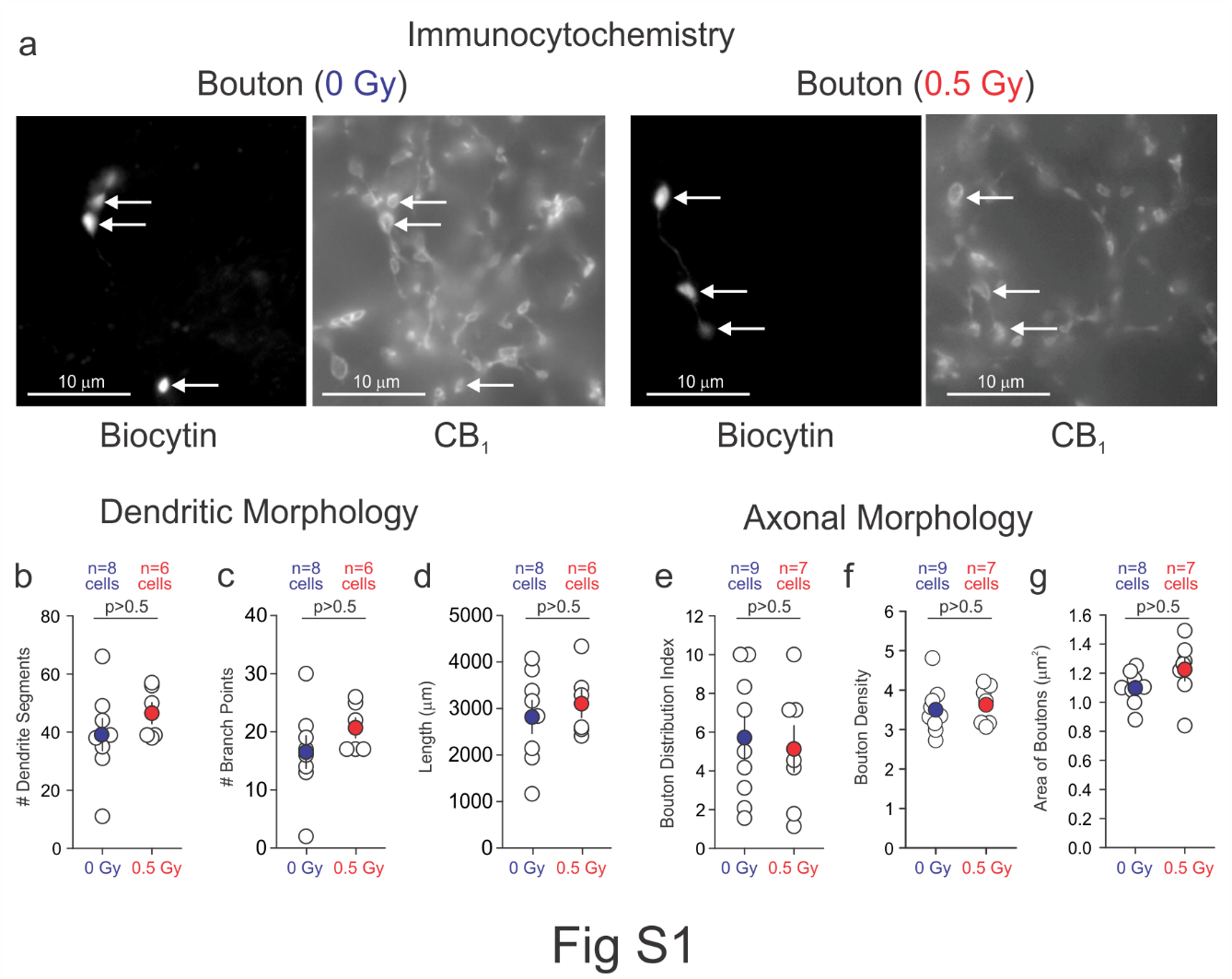
**

**
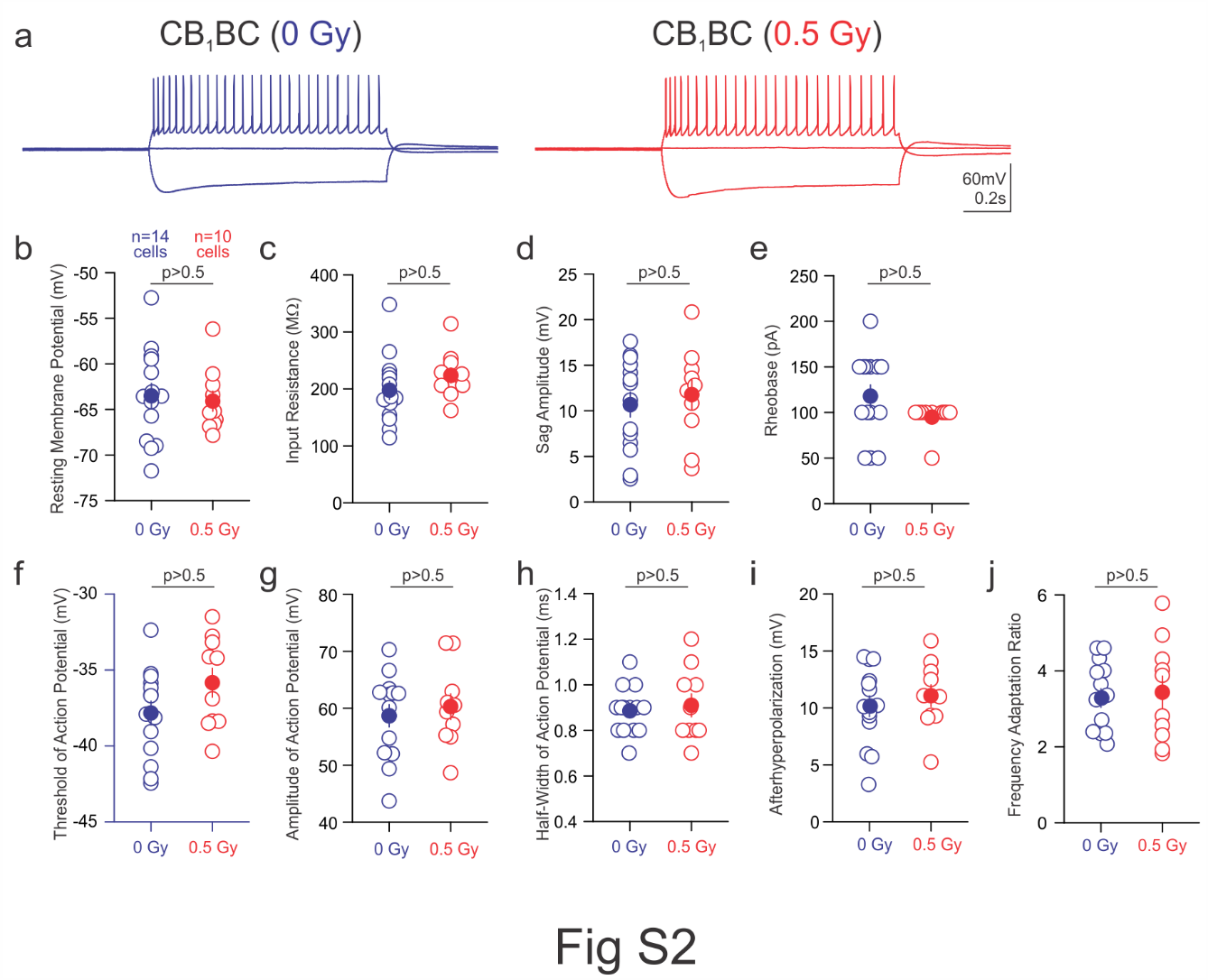
**

**
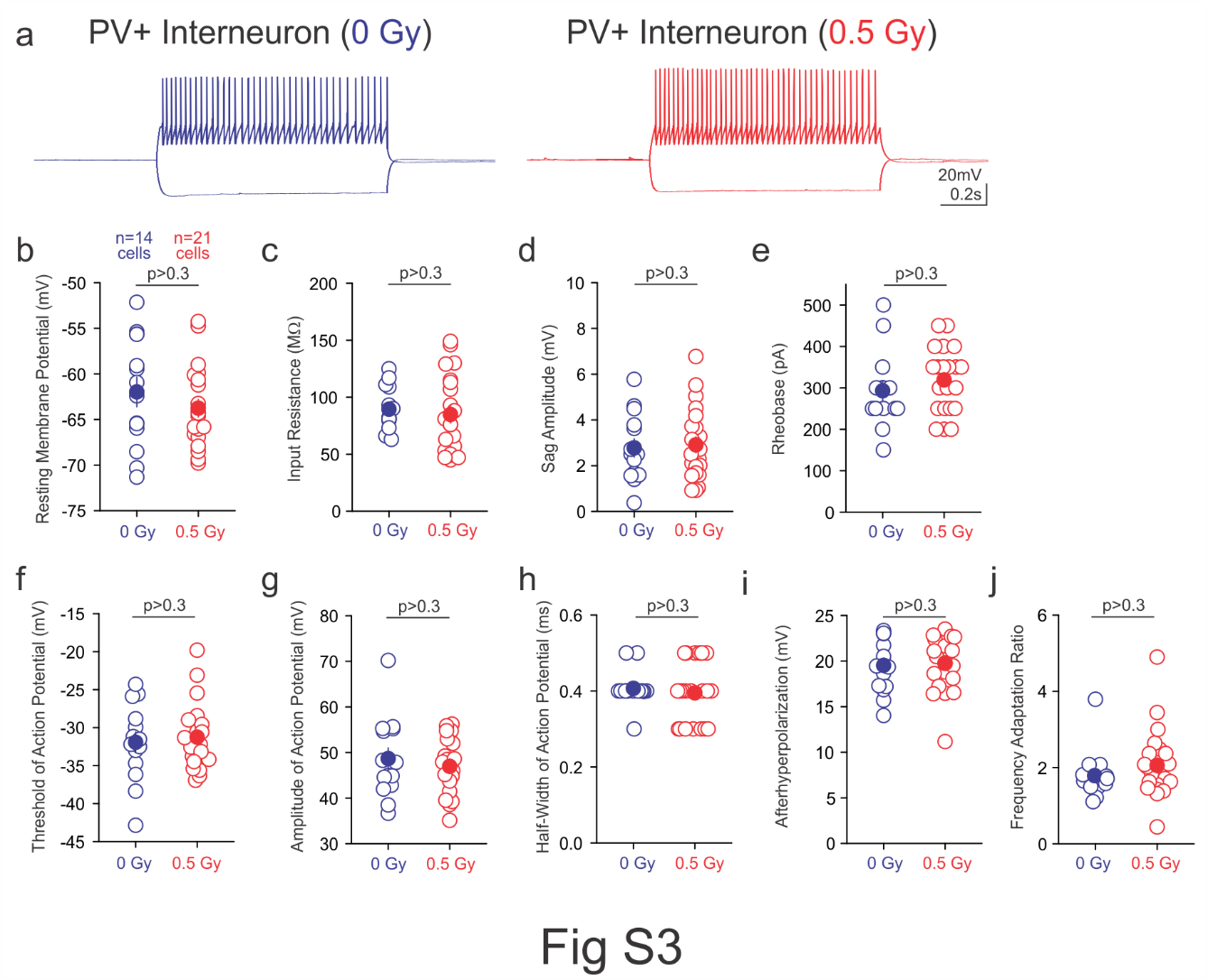
**

**Fig S1** In CB_1_BCs, irradiation does not alter expression of CB_1_ or neuronal morphology. **a** Double-immunofluorescence staining of CB_1_ and biocytin was not changed in the axon terminals (labeled by arrows) of CB_1_BCs. **b**−**d** Dendritic morphology of CB_1_BCs is also unchanged by irradiation. Summary plots of segment number (**b**), number of branch points (**c**), and length (**d**). **e**−**g** Axonal morphology. Summary plots of bouton distribution index (**e**), bouton density (**f**), and area of boutons (**g**). Open circles represent values for individual cells. Blue or red filled circles label averages in all supplemental figures.

**Fig S2** Irradiation does not alter the intrinsic properties of CB_1_BCs. **a** Example voltage traces of CB_1_BCs in response to depolarizing and hyperpolarizing current steps (200 and -300pA, respectively, from -65mV). **b**−**j** Summary plots demonstrating that irradiation does not alter the intrinsic properties of CB_1_BCs including: resting membrane potential (**b**), input resistance (**c**), sag amplitude (**d**), rheobase (**e**), AP threshold (**f**), AP amplitude (**g**), AP half-width (**h**), AHP amplitude (**i**), and frequency adaptation ratio (**j**).

**Fig S3** Irradiation does not alter the intrinsic properties of PVINs. **a** Example voltage traces of PVINs in response to depolarizing and hyperpolarizing current steps (350 and -300pA, respectively, from -65mV). **b**−**j** Summary plots demonstrating that irradiation does not alter the intrinsic properties of PVINs including: resting membrane potential (**b**), input resistance (**c**), sag amplitude (**d**), rheobase (**e**), AP threshold (**f**), AP amplitude (**g**), AP half-width (**h**), AHP amplitude (**i**), and frequency adaptation ratio (**j**).
